# UBC9-mediated SUMOylation of CORO1C drives lung adenocarcinoma progression via Arp2/3-dependent cytoskeletal remodeling

**DOI:** 10.1038/s41419-026-08653-w

**Published:** 2026-03-30

**Authors:** Zhe Zhang, Bing Xiao, Yupeng Jiang, Lixia Niu, Li Wang, Juan Cai

**Affiliations:** 1 Department of Thoracic Surgery, The Second Xiangya Hospital, Central South University, Changsha, China; 2 Thoracic Surgery Research Laboratory, The Second Xiangya Hospital, Central South University, Changsha, China; 3 Hunan Key Laboratory of Early Diagnosis and Precise Treatment of Lung Cancer, The Second Xiangya Hospital, Central South University, Changsha, China; 4 National Clinical Research Center for Metabolic Diseases, The Second Xiangya Hospital of Central South University, Changsha, China; 5 Department of Emergency Medicine, The Second Xiangya Hospital, Central South University, Changsha, China; 6 Department of Oncology, The Second Xiangya Hospital, Central South University, Changsha, China; 7 Hunan Key Laboratory of Kidney Disease and Blood Purification, Department of Nephrology, The Second Xiangya Hospital at Central South University, Changsha, China

**Keywords:** Non-small-cell lung cancer, Sumoylation

## Abstract

Lung adenocarcinoma (LUAD) remains a leading cause of cancer-related mortality worldwide, and understanding its molecular drivers is critical for improving therapeutic outcomes. SUMOylation is a form of post-translational modification that conjugates small ubiquitin-like modifier (SUMO) to specific lysine residues of target proteins. The sole SUMO E2-conjugating enzyme UBC9 is upregulated in multiple malignancies, yet its functional role and specific substrate in LUAD remain poorly defined. Here, we demonstrate that UBC9 is significantly elevated in LUAD tissues, and its high expression is associated with adverse clinic outcomes. Functional assays revealed that genetic ablation of UBC9 robustly suppresses LUAD cell proliferation, migration, invasion, and tumorigenesis both in vitro and in vivo. Through immunoprecipitation-mass spectrometry, we identified Coronin-1C (CORO1C), a master regulator of actin dynamics, as a key substrate of UBC9-mediated SUMOylation. Mechanistically, SUMOylation of CORO1C at lysine residues K19, K311, and K440 enhances its binding to actin-related protein 2 (Arp2) complex, promotes actin‑based cytoskeletal remodeling, and drives malignant cellular behaviors. Collectively, our work reveals a previously unrecognized regulatory axis in which UBC9‑dependent SUMOylation licenses CORO1C to orchestrate Arp2/3‑mediated cytoskeletal dynamics, thereby advancing LUAD progression. These findings reveal CORO1C as a novel SUMOylation target in lung adenocarcinoma and offer new mechanistic insights into tumor cell motility, but also highlight a promising therapeutic avenue for treating advanced LUAD.

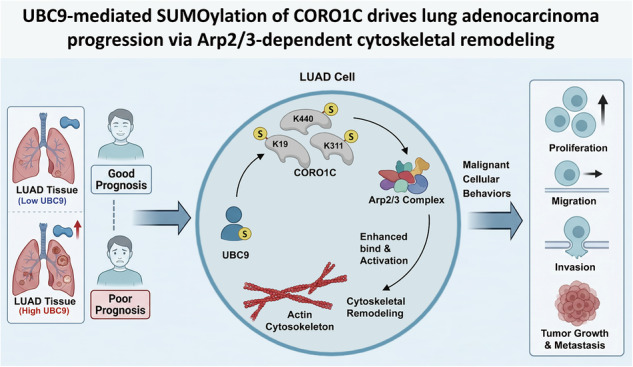

## Introduction

Lung cancer represents the foremost cause of cancer-related mortality worldwide, accounting for approximately 18% of total cancer deaths according to recent global estimates [1, 2]. Histologically, it is broadly categorized into small cell lung cancer (SCLC) and non-small cell lung cancer (NSCLC), with NSCLC comprising about 85% of all cases [3, 4]. Among NSCLC subtypes, lung adenocarcinoma (LUAD) is the most prevalent form, characterized by distinct pathological and molecular features [5]. Despite significant advancements in clinical management, including the development of targeted therapies such as tyrosine kinase inhibitors, the prognosis for LUAD patients remains suboptimal, with a persistently modest 5-year survival rate [6, 7]. This poor prognosis underscores the urgent need to elucidate the underlying molecular mechanisms driving LUAD progression.

Post-translational modifications (PTMs) are essential regulators of diverse cellular processes, modulating protein-protein interactions, transcriptional regulation, subcellular trafficking, and protein stability [8]. Among these, SUMOylation, a dynamic and reversible covalent attachment of Small Ubiquitin-like Modifier (SUMO) proteins to target substrates, has garnered increasing attention for its pivotal role in oncogenesis [9]. The SUMOylation cascade is orchestrated by E1-activating enzymes SAE1/SAE2, E2-conjugating enzyme UBC9 (coding by the *UBE2I* gene), and E3 ligases [10–12]. Conversely, de-SUMOylation is mediated by a family of Sentrin/SUMO-specific proteases (SENPs), with SENP1 being the principal de-SUMOylated protease in mammalian cells [13]. UBC9, as the sole E2 enzyme, is indispensable for SUMOylation, and its dysregulation has been implicated in various malignancies. For instance, elevated UBC9 expression correlates with tumor grade and poor prognosis in breast cancer, where it modulates cellular migration and invasion [14–16]. Similarly, UBC9 is overexpressed in gastric cancer tissues and contributes to tumor progression [17]. Notably, elevated UBC9 expression has been reported in primary lung cancer tissues and metastatic lesions, and its overexpression promotes migratory and invasive phenotypes in lung cancer models [18]. However, the precise functional roles, regulatory mechanisms, and pathological significance of UBC9 specifically in LUAD remain poorly characterized.

Cytoskeletal dynamics and cell motility are fundamental to cancer progression and metastasis [19, 20], processes critically dependent on actin polymerization and membrane protrusion formation [21, 22]. The actin-related protein 2/3 (Arp2/3) complex serves as a central nucleator of branched actin networks, driving lamellipodia formation and directional cell migration [23]. Coronin-1C (CORO1C), a member of the coronin family, directly binds to and regulates the Arp2/3 complex, thereby modulating actin filament assembly and cell motility [24–26]. Accumulating evidence indicates that CORO1C is upregulated in multiple cancer types and promotes malignant phenotypes, including enhanced migration, invasion, and metastasis [27, 28]. Its activity is known to be regulated by PTM, such as phosphorylation [29]. Nevertheless, the role of CORO1C in LUAD pathogenesis, along with its upstream regulatory mechanisms, particularly through SUMOylation, remains largely unexplored. Whether and how UBC9-mediated SUMOylation influences CORO1C function to drive LUAD progression represents a significant knowledge gap.

In this study, we systematically demonstrate that UBC9 is significantly upregulated in LUAD and its high expression predicts poor prognosis. Functionally, genetic ablation of UBC9 suppresses the proliferation, migration, invasion, and tumorigenic capacity of LUAD cells both in vitro and in vivo. Through immunoprecipitation-mass spectrometry (IP-MS) analysis, we identify CORO1C as a key substrate of UBC9-mediated SUMOylation. Importantly, SUMOylation at three specific lysine residues (K19, K311, and K440) is indispensable for CORO1C to interact with Arp2, driving subsequent actin cytoskeletal remodeling and the execution of its pro-tumorigenic phenotypes. Collectively, our findings not only establish CORO1C as a novel SUMOylated effector in LUAD but also delineate a functional axis wherein UBC9-mediated SUMOylation licenses CORO1C to activate the Arp2/3 complex, thereby promoting cytoskeletal dynamics and malignant progression. This work not only offers new mechanistic insights into tumor cell motility but also highlights a promising therapeutic avenue for treating advanced LUAD.

## Materials and methods

### Cell culture and treatment

All cell lines used in this study were purchased from the American Type Culture Collection (ATCC). Human bronchial epithelial cells BEAS-2B and embryonic kidney cells 293T were cultured in DMEM (Dulbecco’s modified Eagle medium). All lung adenocarcinoma (LUAD) cell lines (H23, H1299, H1975, HCC827, A549, PC9) were cultured in RPMI-1640 medium. All media were supplemented with 10% fetal bovine serum and 1% penicillin–streptomycin. Cells were incubated at 37 °C in a humidified 5% CO₂ atmosphere. For pharmacological inhibition of the Arp2/3 complex, cells were treated with CK-666 (MCE, #HY-16926) at a final concentration of 100 μM.

### Patient tissue specimens

Paired LUAD tumor and adjacent non-tumor tissues were obtained from patients undergoing surgical resection at the Second Xiangya Hospital, Central South University, between 2020 and 2023. Written informed consent was obtained from all participants. The study was conducted in accordance with the Declaration of Helsinki and approved by the Institutional Ethics Committee (Ethics approval No. LYEC2024-0033).

### Bioinformatics analysis

Gene Expression Profiling Interactive Analysis (GEPIA, http://gepia.cancer-pku.cn/) was utilized to analyze UBC9 mRNA expression across various tumor cohorts. Specifically, UBC9 expression in LUAD versus adjacent normal tissues, as well as its correlation with overall survival (OS) and disease-free survival (DFS) in LUAD patients, were evaluated.

### Plasmids, lentiviral production, and stable cell line generation

Expression plasmids for HA-SENP1, His-SUMO1, Myc-UBC9, Flag-CORO1C (WT and the indicated mutant), and UBC9-targeting sgRNAs (cloned into lentiCRISPRv2) were obtained from Tsingke Biotechnology (China). Lentiviruses were packaged in 293T cells using psPAX2 and pMD2.G with Lipofectamine 2000 (Invitrogen, #11668019). Viral supernatants were collected 24–48 h post‑transfection, filtered (0.22 μm), and used to infect target cells in the presence of 2 μg/mL polybrene (Beyotime, #C0351). Stable cells were selected with 5 μg/mL puromycin (Beyotime, #ST551) for 7 days. The sequences of sgRNA and CORO1C SUMOylation mutants were listed in Supplementary Table S1.

### Protein extraction and Western blotting

Cells and tissues were lysed in RIPA buffer (Beyotime, #P0013B) supplemented with a protease inhibitor cocktail. Protein concentrations were determined using the BCA assay. Equal amounts of protein (30 μg) were separated by SDS-PAGE and transferred onto a PVDF membrane (Millipore). The membrane was blocked with 5% non-fat milk in TBST for 1 h at room temperature, followed by incubation with primary antibodies overnight at 4 °C. After washing, the membrane was incubated with HRP-conjugated secondary antibodies for 1 h at room temperature. Protein signals were visualized using Super ECL Plus reagent (UE, #S6009L) and captured with a SageCapture™ MiniChemi610 imaging system. Antibody details are provided in Supplementary Table S2.

### Immunoprecipitation and liquid chromatograph mass spectrometer (LC–MS/MS) analysis

Cells (2 × 10⁷) were lysed in RIPA buffer with protease inhibitors. Cleared lysates were incubated with specific antibodies or control IgG overnight at 4 °C, followed by protein A/G magnetic beads (#P2108, Beyotime) at room temperature for 3 h. Beads were washed with TBST and subsequently sent to OE Biotech Co., Ltd. (Shanghai, China) for LC-MS/MS analysis. For protein interaction analysis, the beads were resuspended in 1x SDS loading buffer and heated at 95 °C for 10 min, followed by western blot analysis to detect co‑immunoprecipitated proteins. For the SUMOylation assay, 293T cells were co-transfected with Flag-CORO1C or the indicated mutant, Myc-UBC9, His-SUMO1, and/or HA-SENP1. After 48 h of transfection, the supernatant of cell lysis was incubated with flag-magnetic beads (#20565ES03, Yeasen) overnight at 4 °C. The beads were washed three times with TBST, resuspended in 1x SDS loading buffer, and analyzed by Western blot.

### Cell counting kit-8 (CCK8) assay

Cell viability was determined using the Cell Counting Kit-8 (CCK‑8) assay. Briefly, cells were seeded at a density of 5 × 10³ cells per well in 96‑well plates. At indicated time points, CCK‑8 reagent was added to each well, followed by incubation for 3 h at 37 °C. Absorbance was measured at 450 nm using a microplate reader.

### Colony formation assay

A549 and PC9 cells were trypsinized, counted, and resuspended in serum-free medium. Subsequently, cells were seeded into 6‑well plates at a density of 1 × 10³ cells per well for 14 days. Colonies were then fixed with 4% paraformaldehyde for 15 min, stained with 0.1% crystal violet for 10 min, and imaged. The number of colonies was quantified using FIJI software.

### Transwell migration and invasion assays

Cell migration and invasion were evaluated using Transwell chambers (Corning). Prior to the assay, cells were serum‑starved for 24 h.

#### Migration assay

A suspension of 4 × 10⁴ cells in 200 µL of serum-free medium was added to the upper chamber. The lower chamber was filled with 500 µL of complete medium containing 10% FBS as a chemoattractant. After a 24-h incubation at 37 °C, non-migrated cells on the upper surface of the membrane were gently removed with a cotton swab. The migrated cells on the lower surface were fixed with 4% paraformaldehyde, stained with crystal violet, and imaged. The number of cells in five randomly selected fields per membrane was counted using FIJI software.

#### Invasion assay

The invasion assay was performed identically to the migration assay, except that the Transwell inserts were pre-coated with Matrigel (diluted 1:8 in serum-free medium) to simulate the extracellular matrix. A suspension of 8 × 10⁴ cells in 200 µL of serum-free medium was seeded into the Matrigel-coated upper chambers. Subsequent incubation, fixation, staining, and quantification steps were identical to those described for the migration assay.

### Immunohistochemistry (IHC) and immunofluorescence (IF)

Formalin-fixed, paraffin-embedded tissues were sectioned and stained for UBC9 using standard IHC protocols (BIOSSCI Biotechnology, China). For immunofluorescence, cells were fixed with 4% PFA, permeabilized with 0.3% Triton X-100, blocked with 5% BSA, and incubated with primary antibodies overnight at 4 °C, followed by fluorophore-conjugated secondary antibodies. Nuclei were counterstained with DAPI. Images were acquired using a confocal microscope, and the co-localization of ARP2 and Flag-CORO1C WT or 3KR mutant was analyzed using the Plot Profile function in FIJI software to calculate the coefficient (MOC). For each biological replicate (*n* = 3), 10 randomly selected, non-overlapping fields of view were quantified. The data presented in the figures represent the mean MOC values derived from these independent biological replicates. For F-actin visualization, fixed cells were stained with Alexa Fluor-conjugated phalloidin (Proteintech, #PF00003, 1:200) for 20 min at 37 °C. Images were acquired using a super-resolution microscope (CSR Biotech HIS-SIM). Antibody details are provided in Table S2.

### Animal studies

All animal procedures were approved by the Experimental Animal Ethics Committee of the Second Xiangya Hospital, Central South University (Approval No. 2020377). Four to six-week-old male BALB/c nude mice were used.

For the subcutaneous xenograft model, a total of 5 × 10^6^ cells in 100 μL PBS were injected subcutaneously into the right flank of each mouse. Tumor length and width were measured every 5 days with a caliper, and volume (mm³) was calculated as π/6 × length × width².

For the experimental lung metastasis model, mice were injected intravenously with 5 × 10^5^ cells in 100 μL PBS. For CK-666 treatment, mice received intraperitoneal injections of CK-666 (20 mg/kg) once weekly, starting two weeks post-cell injection. After 8 weeks, mice were euthanized, and lungs were harvested for H&E staining and quantification of metastatic nodules. For the lung metastasis analysis, the statistical unit was defined as the number of metastatic nodules per whole lung. Quantification was performed on five independent biological replicates.

For in vivo bioluminescence imaging, mice bearing subcutaneous tumors derived from luciferase-expressing cells were intraperitoneally injected with D-luciferin (Aladdin, # L120798, 150 mg/kg). Anesthetized mice were imaged 15 min later using the 3D Live Animal Imaging System (PerkinElmer, IVIS Spectrum).

### Statistical analysis

All in vitro experiments were performed in at least three independent replicates. Statistical analyses were conducted using GraphPad Prism 10.4.1. Data are presented as mean ± standard deviation (SD). Differences between the two groups were analyzed using an unpaired two-tailed Student’s *t* test. The differential analysis of multiple groups was performed using one-way analysis of variance (ANOVA) and two-way ANOVA followed by Dunnett’s test. A *p*-value < 0.05 was considered statistically significant.

## Results

### UBC9 is significantly upregulated in LUAD tissues associated with adverse prognosis

**Fig. 1:**
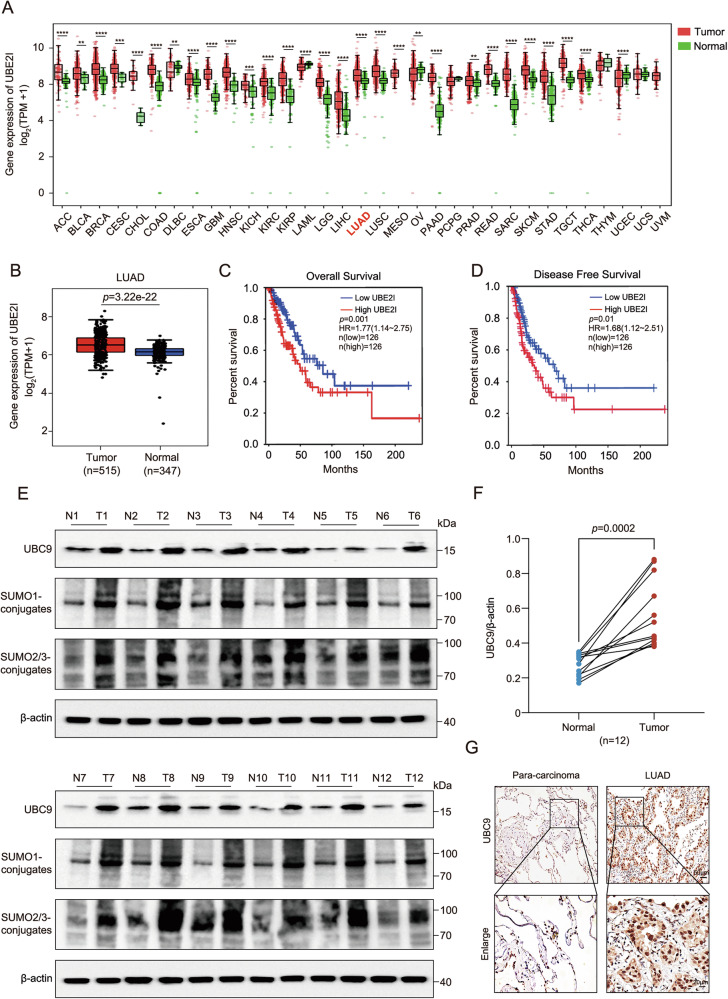
UBC9 is highly expressed in lung adenocarcinoma (LUAD) and correlates with poor prognosis. Analysis of the GEPIA (http://www.gepia.cancer-pku.cn/) database. **A** Pan-cancer analysis showing UBE2I (UBC9) mRNA expression levels in various human tumor types and the adjacent normal (N) tissues. LUAD is highlighted in red. **B** Expression comparison of UBC9 mRNA between LUAD tissues and adjacent normal tissues. Kaplan–Meier survival analysis of overall survival (OS) (**C**) and disease-free survival (DFS) (**D**) in LUAD patients stratified by high vs. low UBC9 expression. **E** Representative immunoblots of of UBC9, SUMO1, and SUMO2/3 in 12 paired tumor (T) and adjacent normal (N) lung tissues from independent LUAD patients. β-actin served as a loading control. **F** Relative quantitative analysis of UBC9 protein expression by Western blot. β‑actin served as a loading control. **G** Representative immunohistochemical analysis of UBC9 expression in paired LUAD and adjacent non-cancerous tissues. Scale bar, 50 μm. Quantitative data are expressed as means ± SD.**p < 0.01, ***p < 0.001, ****p < 0.0001.

To investigate the clinical relevance of UBC9 in lung adenocarcinoma (LUAD), we first analyzed its expression using the GEPIA database. Pan-cancer analysis revealed that *UBE2I* (the gene encoding UBC9) was significantly upregulated in tumor tissues compared to corresponding normal tissues across multiple malignancies, including LUAD (Fig. 1A, B). We then stratified LUAD patients into high- and low-UBC9 expression groups based on the median value. Kaplan-Meier survival analysis demonstrated that high UBC9 expression was significantly associated with worse overall survival (OS) (Fig. 1C) and disease-free survival (DFS) (Fig. 1D) in LUAD patients. To validate these in silico findings, we assessed UBC9 protein levels in paired cancerous and adjacent non-cancerous tissues from LUAD patients by immunoblot and immunohistochemistry (IHC) analyses. Consistent with the bioinformatics data, UBC9 protein expression was markedly higher in tumor tissues compared to matched normal lung tissues. This upregulation was associated with an increase in global SUMOylated protein conjugates, suggesting enhanced SUMOylation activity in LUAD (Fig. 1E–G). Collectively, these data from both public databases and our patient cohort indicate that UBC9 is highly expressed in LUAD, suggesting that UBC9-mediated SUMOylation may be a critical driver of LUAD progression and a determinant of adverse clinical outcomes.

### UBC9 deficiency suppresses the malignant potential of LUAD cells in vivo and in vitro

**Fig. 2:**
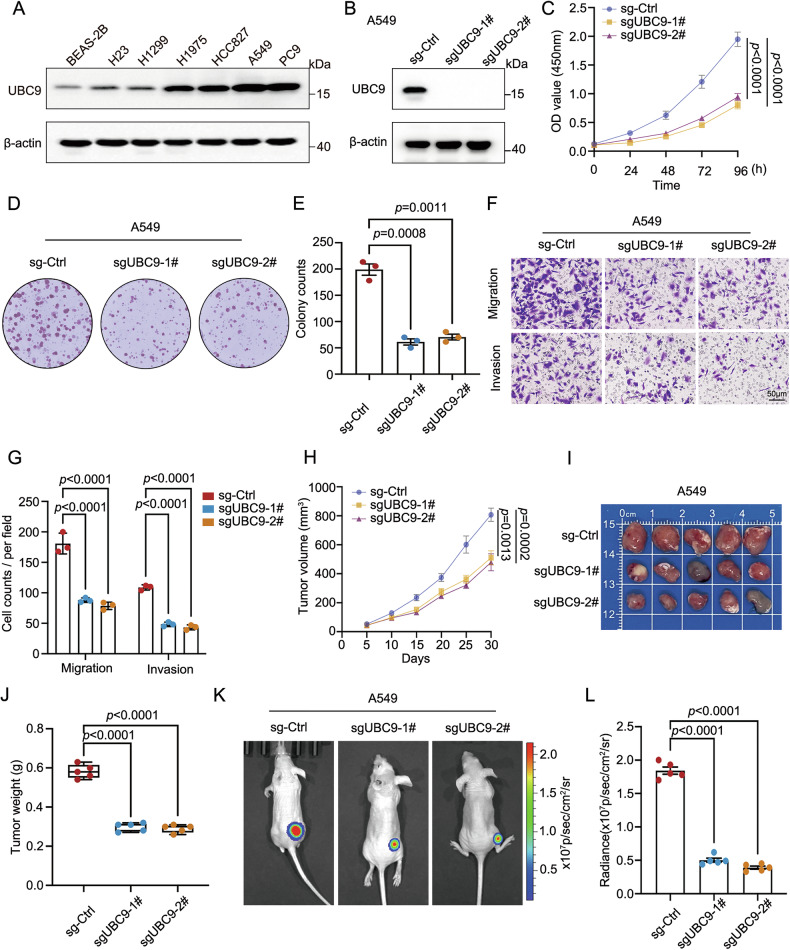
UBC9 knockout impairs the malignant potential of A549 cells. **A** Western blot analysis of UBC9 protein expression in human normal lung epithelial BEAS-2B cells and lung cancer cell lines (H23, H1299, H1975, HCC827, A549, PC9). **B** Western blot validation of UBC9 knockout efficiency in A549 cells using two independent sgRNAs (sgUBC9-1, sgUBC9-2). Assessment of proliferative capacity in sg-Ctrl, sgUBC9-1 and sgUBC9-2 A549 cells by CCK-8 (**C**), colony formation assays (**D**), and quantitative analysis of colony numbers (**E**). **F** Representative images of Transwell migration (upper) and Matrigel invasion (lower) assays in sgCtrl, sgUBC9-1 and sgUBC9-2 A549 cells (scale bar, 50 µm). **G** Quantitative analysis of migrated and invaded cells from (**F**). **H** Tumor growth curves of subcutaneous xenograft tumors derived from sgCtrl, sgUBC9-1, and sgUBC9-2 A549 cells in nude mice (*n* = 5). **I** Photographed dissected tumors from each group at the endpoint (*n* = 5). **J** Final tumor weight in each group. In vivo bioluminescence imaging of orthotopic tumor growth in mice injected with luciferase-expressing sgCtrl or sgUBC9 A549 cells (**K**). **L** Quantitative analysis of in vivo radiance signals in (K). Quantitative data are expressed as means ± SD.

Given the elevated UBC9 in LUAD tissues, we assessed its expression in human normal lung epithelial cell line BEAS-2B (Bronchial Epithelium transformed with Ad12-SV40 2B) and lung cancer cell lines (H23, H1299, H1975, HCC827, A549, PC9). Western blot analysis confirmed that UBC9 protein levels were consistently elevated in all tested LUAD cell lines compared to BEAS-2B, with the highest expression observed in A549 and PC9 cells (Fig. 2A and Supplementary Fig. 1). Consequently, A549 and PC9 cells were selected to generate stable UBC9 knockout cell lines using two distinct sgRNAs. The efficient depletion of UBC9 protein was confirmed by Western blotting (Fig. 2B and Supplementary Fig. 2A). Functional assays demonstrated that UBC9 knockout significantly impaired cell proliferation in A549 or PC9 cells, as measured by CCK-8 assays (Fig. 2C and Supplementary Fig. 2B). This finding was further corroborated by a clonogenic assay, which revealed a marked reduction in the colony-forming ability of UBC9-deficient A549 or PC9 cells compared to control groups (Fig. 2D, E and Supplementary Fig 2C, D). We next evaluated the impact of UBC9 loss on metastatic potential using Transwell migration and Matrigel invasion assays. UBC9 knockout dramatically attenuated both the migratory and invasive abilities of A549 and PC9 cells (Fig. 2F, G and Supplementary Fig. 2E, F). To validate the in vitro findings, we evaluated the impact of UBC9 depletion on tumorigenesis in a xenograft mouse model. A549 cells stably infected with sgCtrl, sgUBC9-1, and sgUBC9-2 were subcutaneously injected into nude mice. In line with the results in vitro, tumors derived from UBC9-knockout cells exhibited significantly slower growth rates compared to the control cohort (Fig. 2H). Final tumor size and weight in the UBC9-deficient groups were substantially reduced (Fig. 2I, J and Supplementary Fig. 2G, H). Additionally, orthotopic tumor progression was further monitored by bioluminescence imaging following implantation of luciferase-expressing A549 and PC9 cells, confirming a significant reduction in tumor burden following UBC9 depletion (Fig. 2K, L and Supplementary Fig. 2I, J). Collectively, these in vitro and in vivo data demonstrate that genetic ablation of UBC9 effectively inhibits the proliferative, migratory, and invasive capacities of LUAD cells.

### UBC9-mediated SUMOylation of CORO1C in LUAD cells

**Fig. 3:**
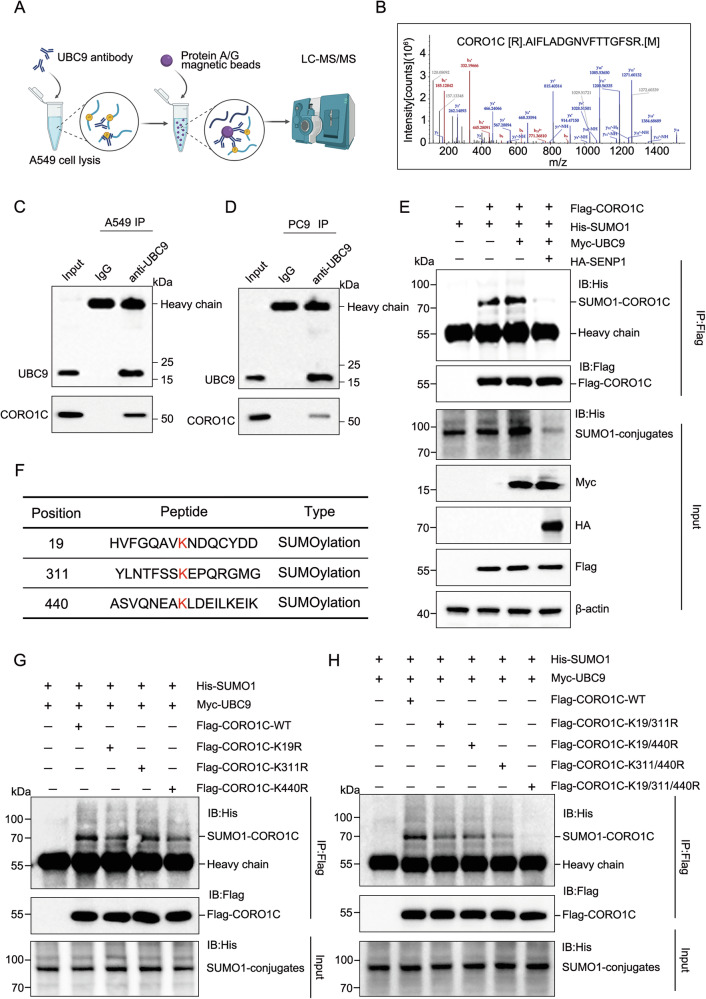
UBC9 mediated the SUMOylation of CORO1C in LUAD cells. **A** Schematic workflow of immunoprecipitation-mass spectrometry (IP-MS) to identify UBC9-binding proteins in A549 cells. **B** Identification of UBC9‑interacting peptides in CORO1C by MS/MS Spectra. Co-immunoprecipitation (Co-IP) assay in A549 (**C**) or PC9 (**D**) cells using anti-UBC9 antibody or IgG control, followed by immunoblotting for UBC9 and CORO1C. **E** HEK293T cells were co-transfected with the indicated plasmids. Cell lysates were immunoprecipitated with FLAG magnetic beads and immunoblotted for His, Myc and Flag. The SUMOylated CORO1C band is indicated. **F** JASSA (http://www.jassa.fr/) predicted the SUMOylation sites of CORO1C. **G** Immunoprecipitation was performed using FLAG magnetic beads to detect the SUMOylation of CORO1C in HEK293T cells co-transfected with Flag-tagged wild-type (WT), K19R, K311R, or K440R, along with His-SUMO1 and myc-UBC9. **H** HEK293T cells were co-transfected with His-SUMO1 and either Flag-tagged wild-type (WT) CORO1C or the indicated lysine-to-arginine mutants. SUMOylation was assessed by IP and immunoblotting.

Given that UBC9 is the sole E2-conjugating enzyme for SUMOylation, we hypothesized that it may facilitate LUAD progression by promoting the SUMOylation of specific substrate proteins. To identify potential substrates, we performed immunoprecipitation coupled with mass spectrometry (IP-MS) using an anti-UBC9 antibody in A549 cells. This analysis identified Coronin-1C (CORO1C) as a prominent UBC9-interacting protein (Fig. 3A, B). CORO1C is an actin-binding protein involved in cytoskeletal remodeling and cell motility. Emerging evidence suggests that CORO1C plays a tumor-promoting role in several cancers by enhancing invasion and metastasis [27, 30]. However, its function and regulatory mechanisms in LUAD remain poorly defined. The interaction between endogenous UBC9 and CORO1C was further validated by co-immunoprecipitation (co-IP) in both A549 and PC9 cells (Fig. 3C, D), suggesting that CORO1C may be a target for SUMOylation. To test this hypothesis, HEK293T cells were co-transfected with plasmids encoding CORO1C and SUMO1. A distinct higher-molecular-weight band migrating approximately 15 kDa above the unmodified CORO1C (corresponding to SUMO1-conjugated CORO1C) was specifically detected, which was absent in cells transfected with SUMO1 alone (Fig. 3E). Importantly, co-overexpression of UBC9 significantly enhanced the intensity of this SUMOylated CORO1C band, whereas co-expression of the deSUMOylase SENP1 eliminated it (Fig. 3E), confirming that CORO1C is a bona fide SUMOylated protein dynamically regulated by the SUMO machinery. We then sought to map the SUMOylation sites on CORO1C. The SUMOylated lysine residue is always within a consensus motif ΨKXD/E (ψ: large hydrophobic residue; K: lysine; X: random amino acid; D: aspartic acid; E: glutamate). Bioinformatic analysis using JASSA (http://www.jassa.fr/) prediction tools identified three putative SUMOylation sites in CORO1C: K19, K311, and K440 (Fig. 3F). We generated a series of CORO1C mutants in which these lysines were substituted with arginine (K-to-R). SUMOylation assays showed that single (K19R, K311R, K440R) and double lysine mutants with various combinations were partially reduced, while the triple mutant (K19R/K311R/K440R, 3KR) of CORO1C was completely abolished, the SUMO1-conjugated band (Fig. 3G, H). Collectively, these results demonstrate that CORO1C is a novel substrate of SUMOylation mediated by UBC9, occurring primarily at lysines 19, 311, or 440.

### SUMOylation is essential for the oncogenic functions of CORO1C in LUAD

**Fig. 4:**
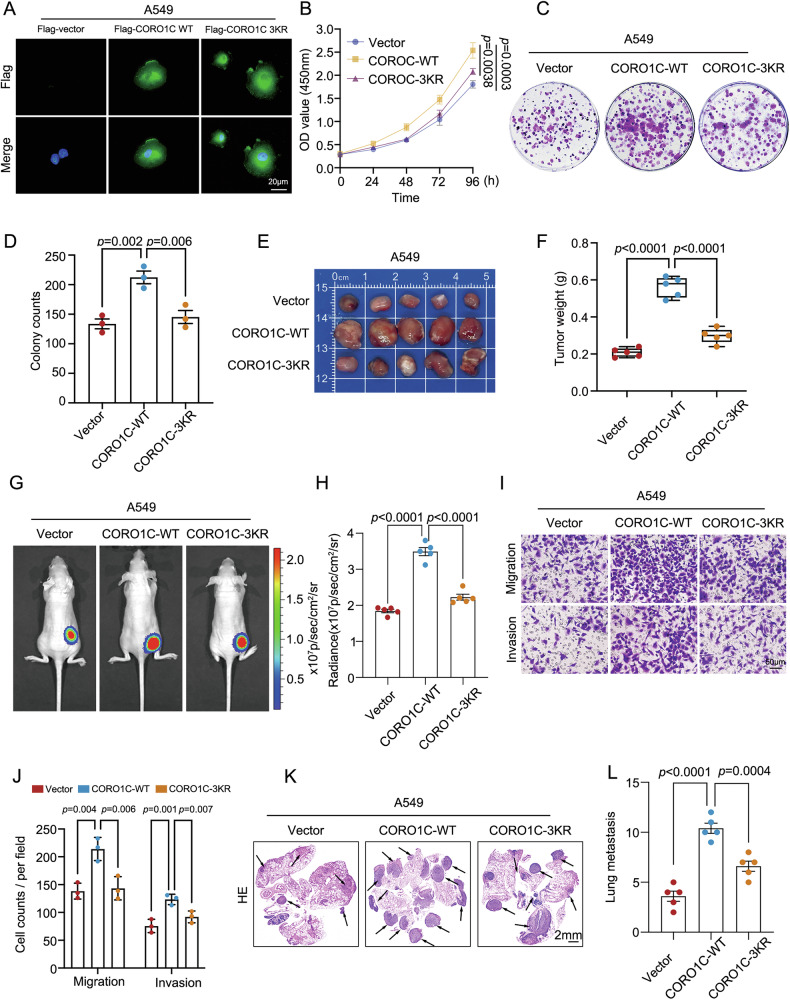
The deSUMOylation status of CORO1C affects the malignant potential of LUAD cells. **A** Immunofluorescence microscopy showing the subcellular localization of CORO1C in A549 cells stably expressing an empty vector, wild-type (WT) CORO1C, or the SUMOylation-deficient 3KR mutant. Nuclei were stained with DAPI. Scale bar, 20 μm. Assessment of proliferative capacity in Flag-Vector, Flag-CORO1C-WT and Flag-CORO1C-3KR A549 cells by CCK-8 assay (**B**) and colony formation assays(**C**), with quantitative statistical analysis of colony numbers (**D**). Subcutaneous xenograft assay in nude mice (n = 5 per group). Final tumor size (**E**) and weight (**F**) were shown. In vivo bioluminescence imaging of an orthotopic lung tumor model. Representative bioluminescent images (**G**) of mice from each group at 8-week post-implantation and quantification of total photon flux (**H**) were shown. Representative images of Transwell migration (upper) and Matrigel invasion (lower) assays. Scale bar: 50μm (**I**). **J** Quantitative analysis of migrated and invaded cells from (**I**). **K** Experimental lung metastasis assay. Representative H&E-stained lung sections from mice injected with the indicated cell lines, with metastatic nodules highlighted by arrows. **L** Quantification of metastatic nodules per lung. Quantitative data are expressed as means ± SD.

The subcellular localization of CORO1C is critical for its role in regulating cytoskeletal dynamics and cell motility. To determine whether SUMOylation affects its distribution, we established A549 stable cell lines expressing empty vector, wild-type (WT) CORO1C or the SUMOylation-deficient 3KR mutant. Immunofluorescence microscopy revealed no significant alteration in their predominant cytoplasmic localization patterns between WT and the 3KR mutant (Fig. 4A). We then investigated the functional impact of CORO1C SUMOylation on tumor progression. In vitro assays revealed that re-expression of WT-CORO1C significantly enhanced cell proliferation and colony formation. However, these pro-proliferative effects were completely abolished by the 3KR mutant (Fig. 4B–D). These findings establish that SUMOylation is indispensable for the pro-proliferative function of CORO1C. These in vitro observations were further validated in vivo. A subcutaneous xenograft assay demonstrated that while WT-CORO1C effectively promoted tumor growth, the 3KR mutant failed to do so (Fig. 4E–F). In vivo bioluminescence imaging further supported these results, showing that tumors derived from WT-CORO1C-expressing A549 cells exhibited significantly higher and more sustained luminescent signals, whereas the 3KR group remained comparable to the vector control (Fig. 4G, H). We next investigated the role of CORO1C SUMOylation in metastatic phenotypes. In Transwell assays, re-expression of WT-CORO1C effectively rescued the migratory and invasive capabilities of A549 cells, an effect that the 3KR mutant completely failed to replicate (Fig. 4I, J). Critically, this functional dichotomy extended to an experimental lung metastasis model. Histological analysis of lung sections revealed a substantial number of metastatic nodules in mice injected with WT-CORO1C-expressing cells, a stark contrast to the minimal metastasis observed in the 3KR and vector control groups (Fig. 4K, L). Collectively, these data provide compelling evidence that the SUMOylation of CORO1C is a critical molecular switch that drives the proliferative and metastatic potential of LUAD cells.

### SUMOylation of CORO1C enhances its interaction with Arp2 to promote actin cytoskeletal remodeling

**Fig. 5:**
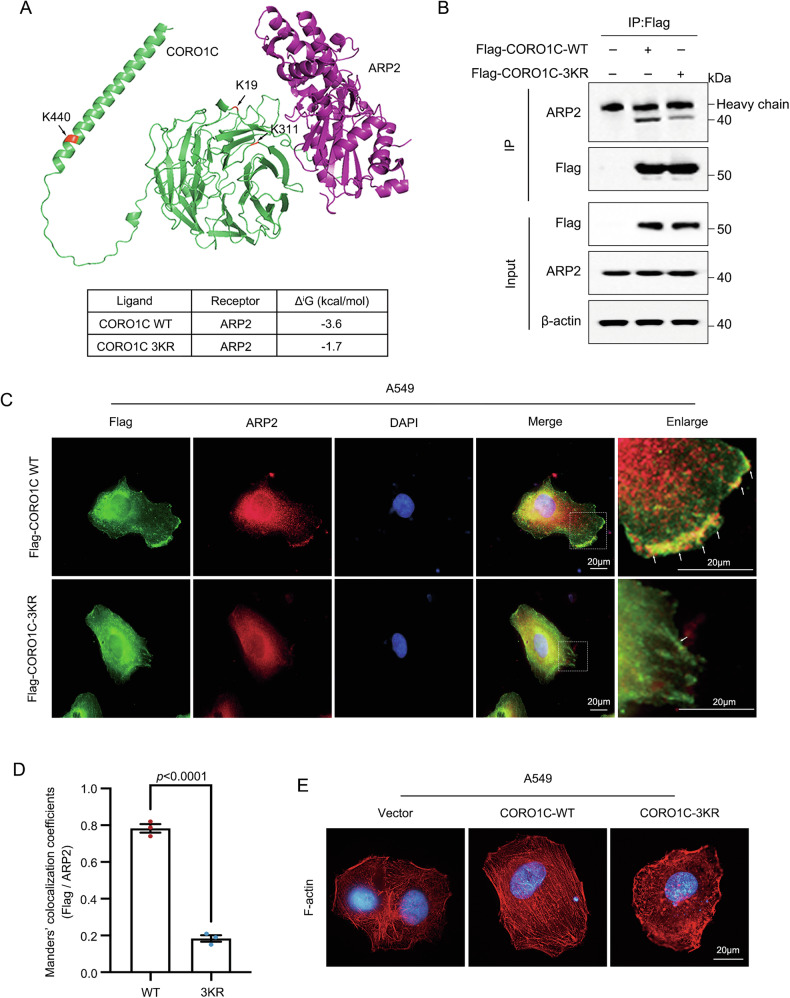
SUMOylation of CORO1C promotes its interaction with Arp2 and actin cytoskeletal remodeling. A549 cells were stably expressing empty vector (Vec), Flag-tagged WT-CORO1C, or the 3KR mutant. **A** Molecular docking analysis of WT or 3KR CORO1C binding to ARP2. **B** Immunoprecipitation was performed using FLAG magnetic beads after cell lysis. Immunoblots detected the expression of Flag and ARP2. **C** Representative immunofluorescence images showing co-localization of flag-tagged WT or 3KR CORO1C (green) and ARP2 (red) in A549 cells. The boxed regions are magnified on the right. Arrows indicate areas of strong co-localization with Flag-CORO1C and Arp2. Scale bar, 20 μm. **D** The colocalization of Flag-CORO1C (WT or 3KR) with ARP2 at the cell periphery was quantified using Manders’ overlap coefficient in Image J. For each of the three independent biological replicates (*n* = 3), 10 randomly selected fields of view were analyzed. **E** Phalloidin staining of F-actin (red) in A549 cells expressing Vector, WT-CORO1C, or 3KR-CORO1C. Scale bar, 20 μm.

CORO1C is known to regulate the actin-related protein 2/3 (Arp2/3) complex, a critical nucleator of branched actin filaments that drives the formation of lamellipodia and filopodia, which are essential for cell migration and invasion. We hypothesized that SUMOylation of CORO1C may modulate its functional interaction with the Arp2/3 complex, thereby influencing LUAD progression. To test this, we first performed molecular docking of WT-CORO1C and 3KR-CORO1C with ARP2 (https://www.ebi.ac.uk/pdbe/). The results showed a stronger binding affinity between WT-CORO1C and ARP2 (–3.6 kcal/mol) compared to 3KR-CORO1C and ARP2 (–1.2 kcal/mol), suggesting that the SUMOylation-deficient 3KR mutant affects binding capacity for ARP2 (Fig. 5A). To further validate this finding, we examined the physical association between CORO1C and Arp2 subunit. Co-immunoprecipitation assays revealed that the SUMOylation-deficient 3KR mutant displayed a reduced binding affinity for Arp2 compared to WT-CORO1C in A549 (Fig. 5B). We next assessed whether this altered biochemical interaction affected their subcellular co-localization at functional sites. Immunofluorescence microscopy demonstrated that in cells expressing WT-CORO1C, there was a strong co-localization of CORO1C and Arp2 at the leading edge of the cell, within membrane protrusions resembling lamellipodia. In contrast, the 3KR mutant showed markedly diminished co-localization with Arp2 in these dynamic regions (Fig. 5C, D). Furthermore, staining of filamentous actin (F-actin) with phalloidin demonstrated that cells expressing WT CORO1C exhibited robust, well-organized actin-rich protrusions, indicative of active Arp2/3-mediated actin polymerization. In contrast, expression of the 3KR mutant resulted in significant actin cytoskeleton disassembly and a loss of the characteristic filamentous network (Fig. 5E). Taken together, these findings indicate that SUMOylation is critical for CORO1C to bind and co-localize with Arp2 at the cell leading edge, thereby orchestrating the actin cytoskeletal remodeling in LUAD cells.

### Pharmacological inhibition of the Arp2/3 complex reverses the oncogenic effects of SUMOylated CORO1C

**Fig. 6:**
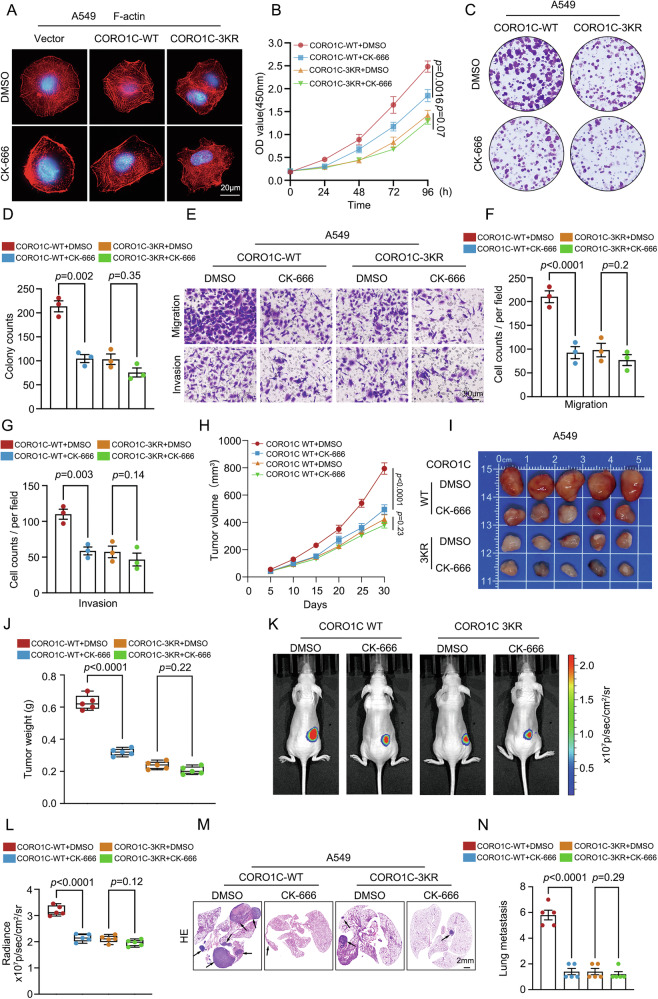
Pharmacological inhibition of the Arp2/3 complex reverses the oncogenic effects of SUMOylated CORO1C. A549 cells reconstituted with WT-CORO1C or 3KR mutant were treated with DMSO or 100 μM CK-666 for 30 h. **A** Representative images of phalloidin-stained F-actin (red) in the indicated cell lines treated with DMSO or CK-666. Nuclei are stained with DAPI (blue). Scale bar, 20 μm. CCK-8 proliferation assay (**B**) and clonogenic assay (**C**) of the indicated cell lines treated with DMSO or CK-666. **D** Quantification of colony numbers from (**C**). **E** Representative images of Transwell migration (upper) and Matrigel invasion (lower) assays after CK-666 treatment. Scale bar, 50 μm. (**F**, **G**) Quantitative analysis of migrated and invaded cells from (**E**). **H** Tumor growth curves of subcutaneous xenografts in mice with or without intraperitoneal injection of CK-666 (20 mg/kg) once a week (*n* = 5). **I** Photographs of dissected tumors from each group at the endpoint (*n* = 5). **J** Final tumor weights in each group. In vivo bioluminescence imaging of orthotopic tumors derived from luciferase-expressing A549 CORO1C WT or 3KR cells in mice with or without intraperitoneal CK-666 treatment. **K** Representative bioluminescence images. **L** Quantitative analysis of in vivo radiance signals in (**K**). Data are expressed as means ± SD. **M** Representative H&E-stained lung sections of metastatic nodules from mice with or without intraperitoneal injection of CK-666 (20 mg/kg) once a week. Arrows indicate metastatic foci. **N** Quantitative analysis of metastatic foci from (**M**). Quantitative data are expressed as means ± SD.

To definitively establish that CORO1C-mediated cytoskeletal remodeling and oncogenic functions are dependent on Arp2/3 complex activation, we employed CK-666, a cell-permeable inhibitor that stabilizes the Arp2/3 complex in an inactive conformation. As shown in Fig. 6A, phalloidin staining revealed that CK-666 treatment effectively disrupted the F-actin network, abolishing the lamellipodial structures induced by WT CORO1C, while exerting minimal effect on the already fragmented and disorganized actin distribution in 3KR-expressing cells. Functionally, CK-666 treatment significantly abrogated the enhanced cell proliferation and colony-forming capacity conferred by WT-CORO1C in A549 cells. In contrast, CK-666 had no significant impact on the already low proliferative and clonogenic potential of A549 cells expressing the 3KR mutant (Fig. 6B–D). Similarly, the migratory and invasive advantages driven by WT-CORO1C were dramatically suppressed upon CK-666 treatment, whereas the motility of 3KR‑expressing cells remained unchanged (Fig. 6E–G). To validate the in vivo relevance of this mechanism, we administered CK-666 intraperitoneally in subcutaneous xenograft and experimental metastasis models. In subcutaneous tumors, CK-666 markedly inhibited the growth of WT CORO1C-expressing tumors, but had no significant effect on 3KR-derived tumors (Fig. 6H–J). Consistent with this, in vivo bioluminescence imaging revealed a pronounced decrease in tumor-associated radiance in the WT group after CK-666 treatment, while the 3KR group showed minimal response (Fig. 6K, L). Crucially, these findings were validated *using* an in vivo lung metastasis model. CK-666 treatment effectively inhibited the metastatic lung colonization-driven by WT-CORO1C. In contrast, the inherently low metastatic potential of 3KR-expressing cells was not further reduced by CK-666 administration (Fig. 6M, N). Collectively, these data demonstrate that the pro-tumorigenic and pro-metastatic functions of SUMOylated CORO1C are mechanistically dependent on its ability to activate the Arp2/3 complex.

## Discussion

**Fig. 7:**
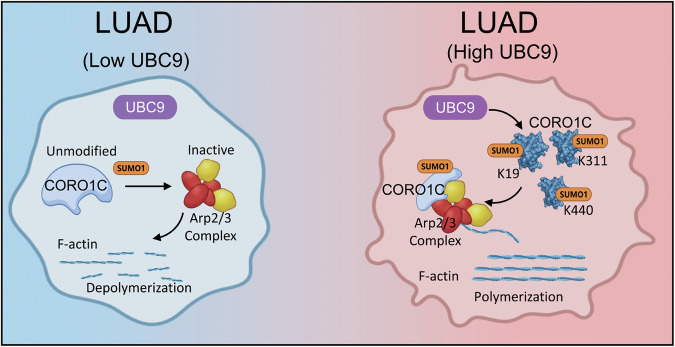
Schematic diagram elucidating the role of UBC9-mediated SUMOylated CORO1C in cytoskeletal remodeling. This schematic illustrates the molecular mechanism by which UBC9 expression influences lung adenocarcinoma (LUAD) progression through modulating the SUMOylation status of CORO1C and subsequent actin dynamics. Left panel (low UBC9 expression): when UBC9 expression is low, CORO1C remains largely unmodified. The unmodified SUMOylated CORO1C exhibits weakened interaction with the Arp2/3 complex, leading to suppressed Arp2/3‑mediated actin polymerization. This cytoskeletal state correlates with inhibited LUAD cell proliferation, migration, invasion, and tumor growth. Right panel (high UBC9 expression): elevated UBC9 expression enhances its activity as the SUMO E2‑conjugating enzyme, promoting site SUMOylation of CORO1C at key lysine residues (K19, K311, and K440). SUMOylated CORO1C shows strengthened binding to the Arp2/3 complex, which drives actin polymerization and cytoskeletal remodeling. This pro‑polymerization state promotes malignant cellular behaviors, including enhanced proliferation, migration, invasion, and tumor growth in LUAD.

Despite significant advances in targeted therapies, lung adenocarcinoma (LUAD) remains a formidable clinical challenge, largely due to an incomplete understanding of its pathogenic mechanisms [31, 32]. Our study illuminates a previously uncharacterized regulatory axis wherein the SUMO-conjugating enzyme UBC9 governs cytoskeletal dynamics to fuel LUAD aggressiveness. We demonstrate that UBC9 achieves this by directly SUMOylation of the actin-binding protein Coronin-1C (CORO1C). These findings elevate SUMOylation as a precise mechanistic switch that directly dictates cytoskeletal organization and, consequently, tumor progression in LUAD (Fig. 7).

While UBC9 overexpression has been reported in several malignancies [33, 34], its functional role in LUAD has remained largely correlative. Here, we establish a direct causal link between UBC9 expression and malignant phenotypes through comprehensive genetic perturbation studies, demonstrating that genetic ablation of UBC9 significantly impairs proliferation, migration, invasion, and tumor growth in vitro and in vivo (Fig. 2 and Supplementary Fig. 2). Importantly, the strong association between high UBC9 expression and poor clinical outcomes further supports its potential as a prognostic biomarker in LUAD (Fig. 1).

A key innovation of this work is the identification of CORO1C as a direct and functionally relevant substrate of UBC9. Using immunoprecipitation-mass spectrometry followed by biochemical validation, we show that CORO1C is robustly SUMOylated in LUAD cells. To our knowledge, this is the first report linking SUMOylation to CORO1C in any cellular context, revealing a previously unrecognized layer of regulation for this cytoskeletal organizer (Fig. 3). Notably, SUMOylation assays detected a single, higher-molecular-weight band (~15 kDa shift) corresponding to mono‑SUMOylated CORO1C. Intriguingly, individual mutation of any of these lysine residues (K19R, K311R, or K440R) did not fully abolish SUMO modification, suggesting that SUMOylation can occur in a flexible, site‑alternating manner when one acceptor site is unavailable. Only the triple lysine‑to‑arginine mutant (3KR) completely abrogated the SUMO‑conjugated band, confirming that these three residues collectively constitute the major, and likely exclusive, SUMO‑acceptor sites on CORO1C.

The dependency of UBC9-driven oncogenicity on CORO1C SUMOylation, which in turn activates Arp2/3-mediated actin remodeling (Fig. 4), suggests that this post-translational modification serves as a critical regulatory node within the cytoskeletal signaling network, and that interfering with downstream actin nucleation could effectively disrupt the pro-tumorigenic cascade initiated by UBC9 (Fig. 5). Moreover, pharmacologic inhibition of Arp2/3 with CK-666 significantly attenuated the proliferation, migration, and metastasis conferred by WT-CORO1C, whereas the SUMOylation-deficient 3KR mutant showed no further suppression with CK-666 (Fig. 6). These data support the central role of Arp2/3, yet we recognize that the reliance on cytoskeletal remodeling is phenotype-dependent. While migration and metastasis are absolutely dependent on Arp2/3-driven actin polymerization, proliferation likely utilizes cytoskeletal dynamics as a permissive factor for cytokinesis and signal transduction, rather than as the sole driver.

Several important questions remain for future investigation. First, the identity of the specific E3 ligase(s) [35, 36] responsible for UBC9-mediated SUMOylation of CORO1C in LUAD remains to be determined. Elucidating this component of the SUMOylation cascade could offer additional targets for pharmacological intervention. Second, it is worth exploring whether the SUMOylation-dependent regulation of CORO1C extends to other coronin family members or functions in other cancer types [37–39], which would indicate a broader role for this modification in cytoskeletal control across malignancies. Additionally, CORO1C activity is known to be regulated by CK2-mediated phosphorylation at serine 463 (S463), which promotes its interaction with Arp2/3 complex, thereby promoting lamellipodia formation [29]. It remains unclear whether SUMOylation and phosphorylation act in concert, sequentially, or antagonistically to fine-tune CORO1C function. Potential mechanisms include conformational changes induced by SUMOylation that facilitate Arp2/3 binding, which may be further modulated by phosphorylation, or phosphorylation-mediated regulation of SUMOylation efficiency at K19, K311, or K440. Future studies aimed at mapping phosphorylation sites on CORO1C in LUAD cells and examining their crosstalk with SUMOylation will be crucial to elucidate the integrated post-translational landscape that governs CORO1C-dependent cytoskeletal remodeling and tumor cell motility.

In summary, this work elucidates a novel UBC9-mediated SUMOylation pathway that coordinates cytoskeletal dynamics to drive the pathogenesis of LUAD. By mechanistically linking SUMOylation to Arp2/3-mediated actin remodeling via CORO1C, we provide a new framework for understanding how post-translational modifications direct cellular architecture in cancer. These insights not only expand the functional repertoire of SUMOylation in oncology but also highlight novel diagnostic and therapeutic opportunities for managing advanced LUAD.

## Supplementary information

Supplementary Table and Figures

supplementary material

checklist

Uncropped image

## Data Availability

The data supporting our findings are available from the corresponding author upon reasonable request.
