# Supplementary material for: UBC9-mediated SUMOylation of CORO1C drives lung adenocarcinoma progression via Arp2/3-dependent cytoskeletal remodeling
Source: Cell Death Dis. 2026 Mar 30;17(1):434. doi: 10.1038/s41419-026-08653-w (PMC13158301; doi:10.1038/s41419-026-08653-w)
Supplement: Supplementary file 1 — Supplementary Table and Figures [file 41419_2026_8653_MOESM1_ESM.docx]

**Supplementary materials**

**UBC9-mediated SUMOylation of CORO1C drives lung adenocarcinoma progression via Arp2/3-dependent cytoskeletal remodeling**

Zhe Zhang^1,2,3^, Bing Xiao^4^, Yupeng Jiang^5^, Lixia Niu^6,7^, Li Wang^1,2,3,*^, Juan Cai^6,7,*^

^1^ Department of Thoracic Surgery, The Second Xiangya Hospital, Central South University

^2^ Thoracic Surgery Research Laboratory, The Second Xiangya Hospital, Central South University

^3^ Hunan Key Laboratory of Early Diagnosis and Precise Treatment of Lung Cancer, The Second Xiangya Hospital, Central South University

^4^ Department of Emergency Medicine, The Second Xiangya Hospital, Central South University

^5^ Department of Oncology, The Second Xiangya Hospital, Central South University

^6^ Department of Nephrology, Hunan Key Laboratory of Kidney Disease and Blood Purification, The Second Xiangya Hospital at Central South University

^7^ National Clinical Research Center for Metabolic Diseases, The Second Xiangya Hospital of Central South University.

***Corresponding author:**

Juan Cai, Department of Nephrology, Hunan Key Laboratory of Kidney Disease and Blood Purification, The Second Xiangya Hospital at Central South University, Changsha 410011, Hunan, China. Changsha 410011, Hunan, China, [cjane218@csu.edu.cn](mailto:cjane218@csu.edu.cn)

Li Wang, Department of Thoracic Surgery, Thoracic Surgery Research Laboratory, Hunan Key Laboratory of Early Diagnosis and Precise Treatment of Lung Cancer, The Second Xiangya Hospital of Central South University, Changsha 410011, Hunan, China, [li-wang@csu.edu.cn](mailto:li-wang@csu.edu.cn)

| Table S1. Primers used for generating sgRNA and SUMOylation mutants of CORO1C | |
| --- | --- |
| UBC9 sgRNA-1# | Forward: 5’-CACCGTATTTCCCCACAGACTCCGT-3’ |
|  | Reverse: 5’-AAACACGGAGTCTGTGGGGAAATAC-3’ |
| UBC9 sgRNA-2# | Forward: 5’-CACCGGCCAGCCATCACAATCAAAC-3’ |
|  | Reverse: 5’-AAACGTTTGATTGTGATGGCTGGCC-3’ |
| K19R CORO1C | Forward: 5’-AGCGGTGCGGAATGACCAGT-3’ |
|  | Reverse: 5’-TCATTCCGCACCGCTTGCCC-3’ |
| K311R CORO1C | Forward: 5’-TCAGCAGCAGGGAGCCTCAGAG-3’ |
|  | Reverse: 5’-GGCTCCCTGCTGCTGAATGTGT-3’ |
| K440R CORO1C | Forward: 5’-ATGAAGCCAGGTTGGATGAGA-3’ |
|  | Reverse: 5’-TCCAACCTGGCTTCATTTTGC-3’ |

| Table S2. Antibody Information | | | | |
| --- | --- | --- | --- | --- |
| **Target** | **Host** | **Manufacturer** | **Application** | **Dilution** |
| UBC9 | Rabbit | #10070-1-AP, Proteintech | WB, IHC | WB: 1:2000  IHC: 1:500 |
| SUMO1 | Rabbit | #10329-1-AP, Proteintech | WB | 1:2000 |
| SUMO2/3 | Mouse | #67154-1-Ig, Proteintech | WB | 1:2000 |
| Flag-M2 | Mouse | # F1804,  Sigma-Aldrich | WB, IF | WB: 1:1000  IF: 1:1000 |
| HA-tag | Rabbit | #81290-1-RR, Proteintech, | WB | 1:10000 |
| His-tag | Rabbit | #66005-1-Ig, Proteintech, | WB | 1:2000 |
| Myc-tag | Rabbit | #60003-2-Ig, Proteintech | WB | 1:2000 |
| beta-actin | Rabbit | #0536-1-AP, Proteintech | WB | 1:2000 |
| CORO1C | Rabbit | #68846-1-Ig, Proteintech | WB | 1:5000 |
| ARP2 | Rabbit | #CY8703, Abways | WB, IF | WB: 1:4000  IF: 1:400 |
| HRP-conjugated goat anti-mouse IgG |  | #SA00001-1, Proteintech | WB | 1:5000 |
| HRP-conjugated goat anti-rabbit IgG |  | #SA00001-2, Proteintech | WB | 1:5000 |
| 488-Goat Anti-Mouse Recombinant Secondary Antibody (H+L) |  | #RGAM002, Proteintech | IF | 1:400 |
| 594-Goat Anti-Rabbit Recombinant Secondary Antibody (H+L) |  | #RGAR004, Proteintech, | IF | 1:400 |

| 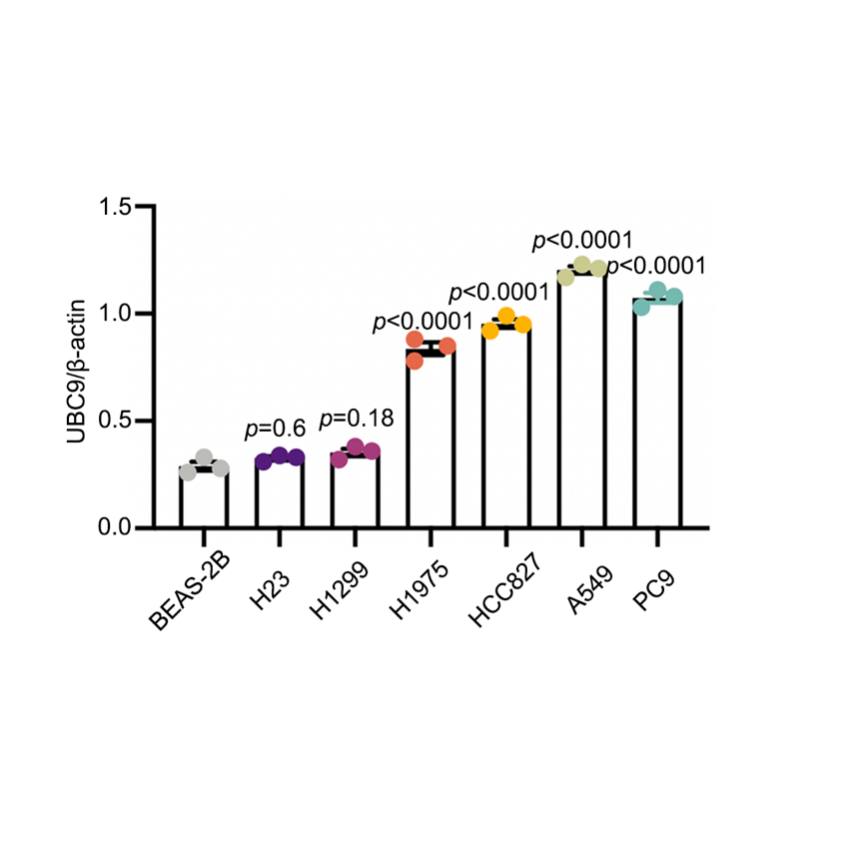 |
| --- |
| Figure S1. Quantitative analysis of UBC9 protein expression normalized to GAPDH from three independent experiments. Data are presented as mean ± SD; statistical significance was determined by one‑way ANOVA. ***p < 0.001 vs. BEAS‑2B. |


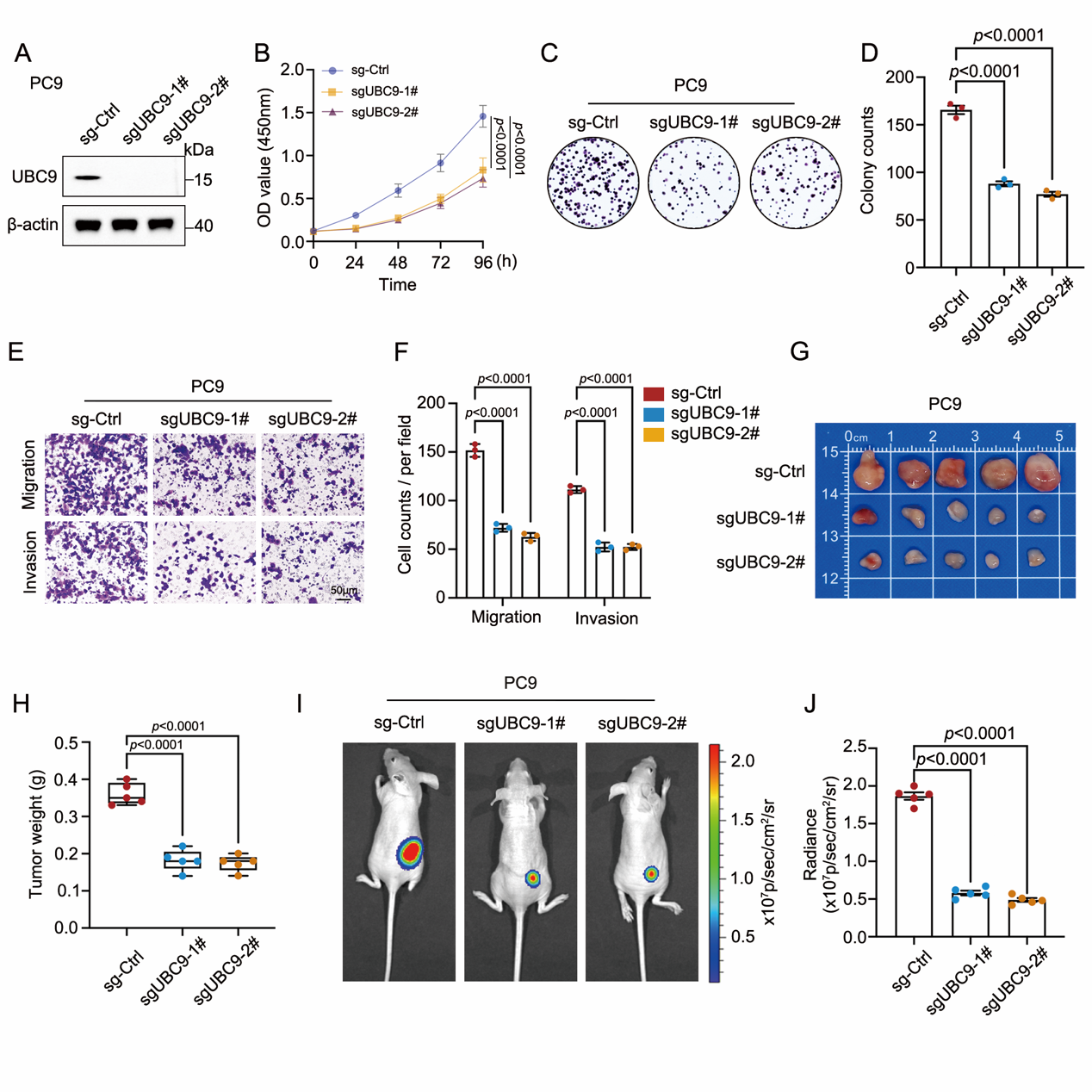


Figure S2. UBC9 deficiency suppresses the proliferation and metastatic potential in PC9 cells. (A) Validation of UBC9 knockout efficiency in PC9 cells stably expressing two independent sgRNAs (sgUBC9-1, sgUBC9-2) or control sgRNA (sgCtrl) by Western blot. (B) CCK-8 assay showing the proliferation curves of PC9 cells with UBC9 knockout. (C-D) Clonogenic assay demonstrating the colony-forming ability of PC9 cells after UBC9 depletion. Representative images (C) and quantification (D) are shown. (E-F) Transwell assays assessing the migration and invasion capabilities of PC9 cells. Representative images of Transwell migration (upper) and Matrigel invasion (lower) assays in sgCtrl and sgUBC9 A549 cells. scale bar, 50µm (E). (F) Quantitative analysis of migrated and invaded cells from (E). (G) Photographs of dissected tumors from each group at the endpoint (n=5). (H) Final tumor weights in each group. (I-J) *In vivo* bioluminescence imaging of orthotopic tumor growth in mice injected with luciferase-expressing sgCtrl- or sgUBC9-PC9 cells (I). (J) Quantitative analysis of *in vivo* radiance signals in (I). Quantitative data are expressed as means ± SD.
