# Supplementary material for: UBC9-mediated SUMOylation of CORO1C drives lung adenocarcinoma progression via Arp2/3-dependent cytoskeletal remodeling
Source: Cell Death Dis. 2026 Mar 30;17(1):434. doi: 10.1038/s41419-026-08653-w (PMC13158301; doi:10.1038/s41419-026-08653-w)

UBC9-mediated SUMOylation of CORO1C drives lung adenocarcinoma progression via Arp2/3-dependent cytoskeletal remodeling

Western Blot original data

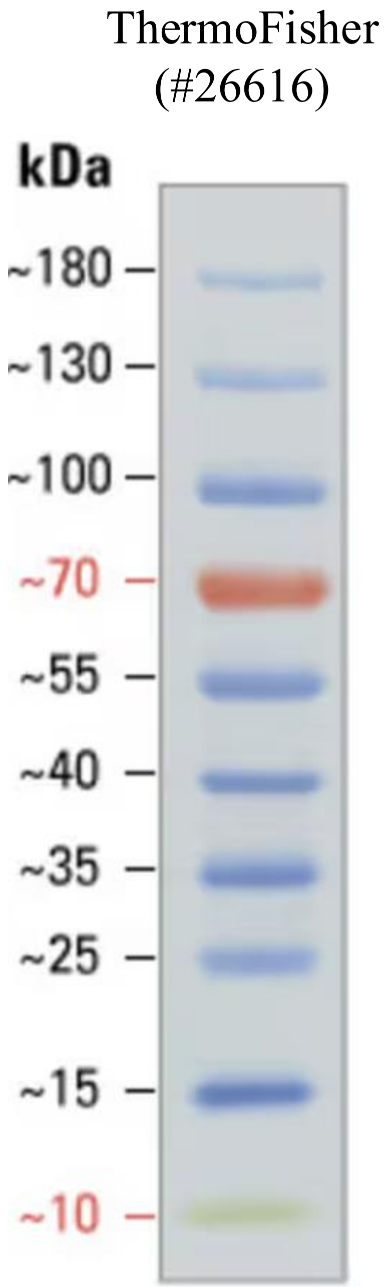
ThermoFisher
(#26616)
kDa
~180
~130
~100
~70
~55
~40
~35
~25
~15
~10

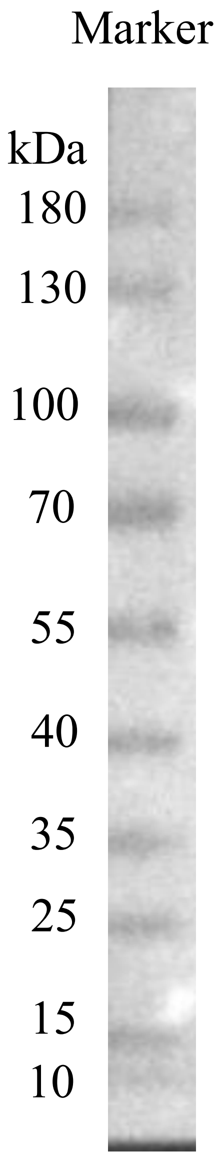
Marker
kDa
180
130
100
70
55
40
35
25
15
10

Figure1E

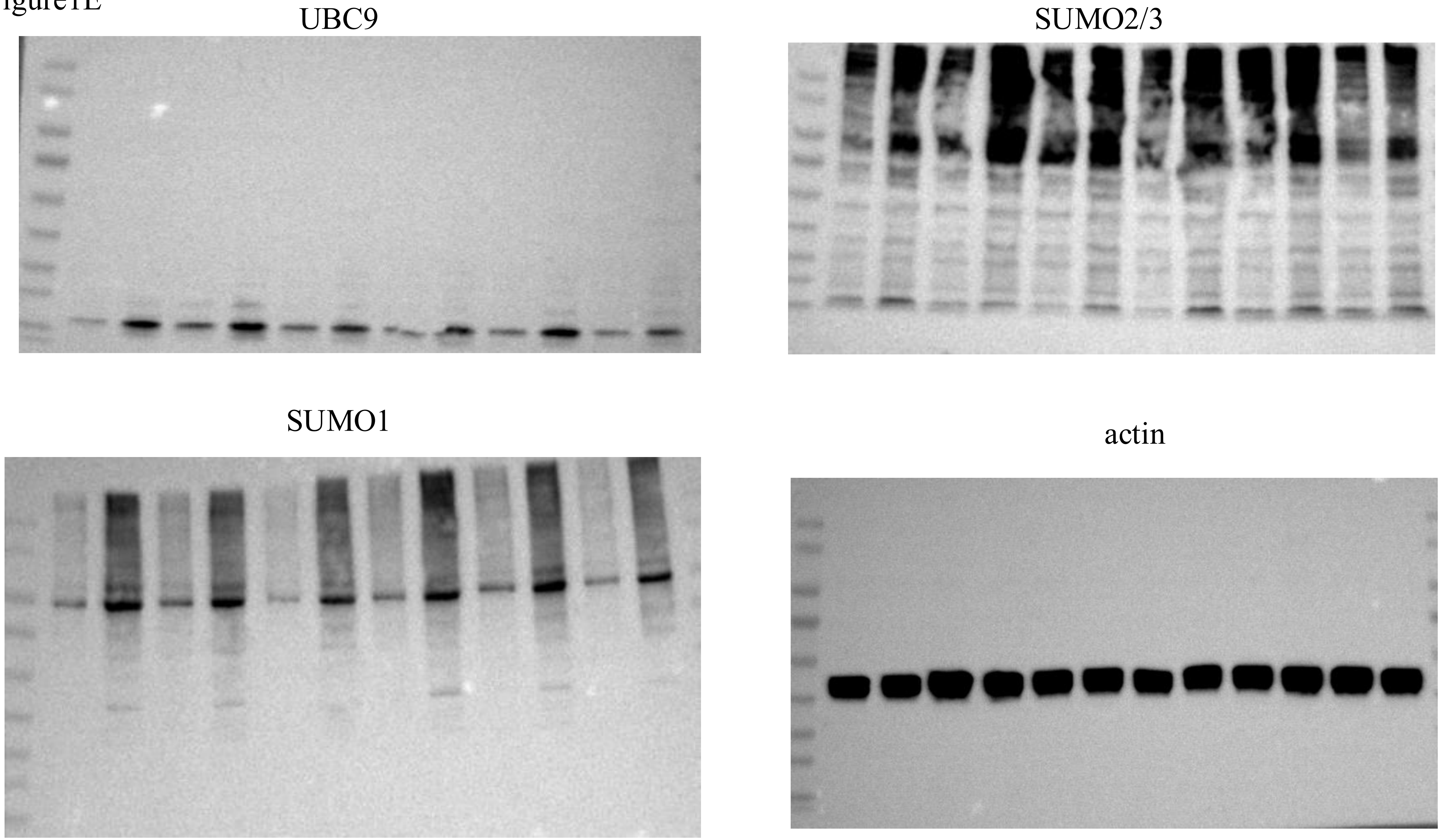

Figure1E

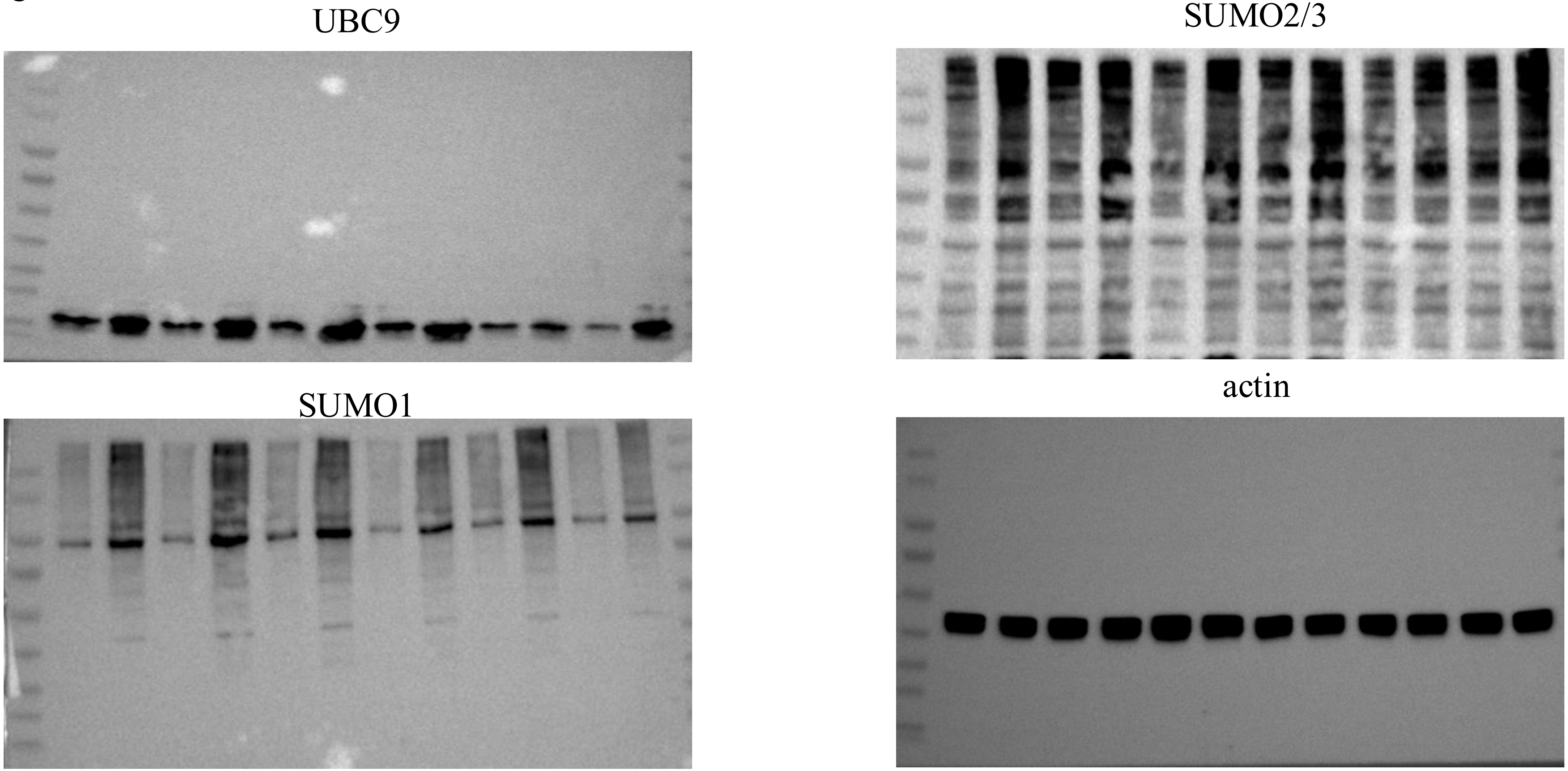

Figure2A

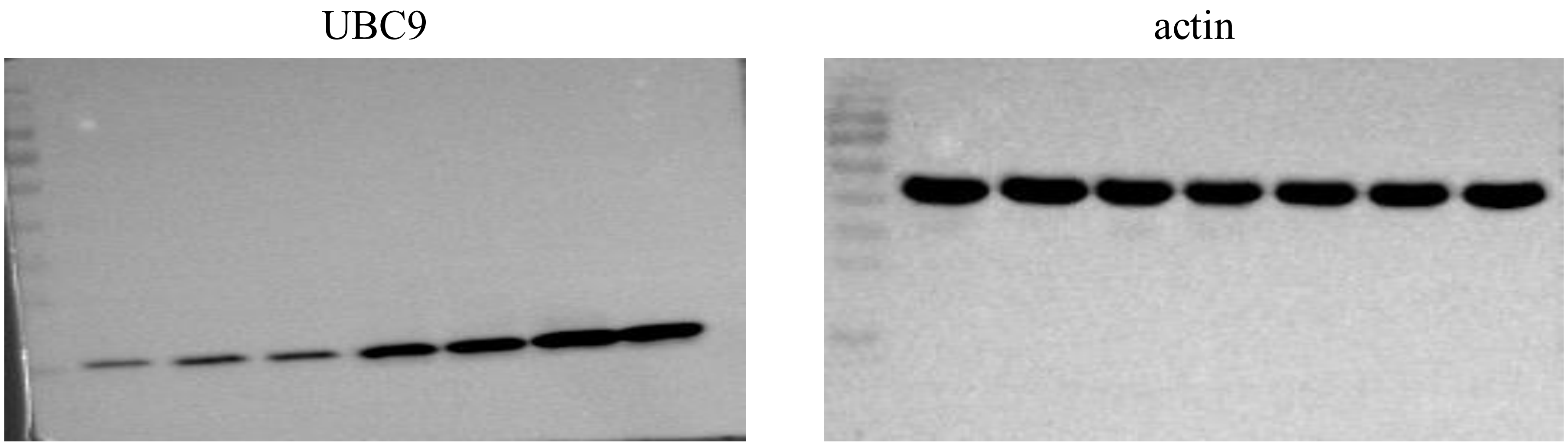

Figure2B

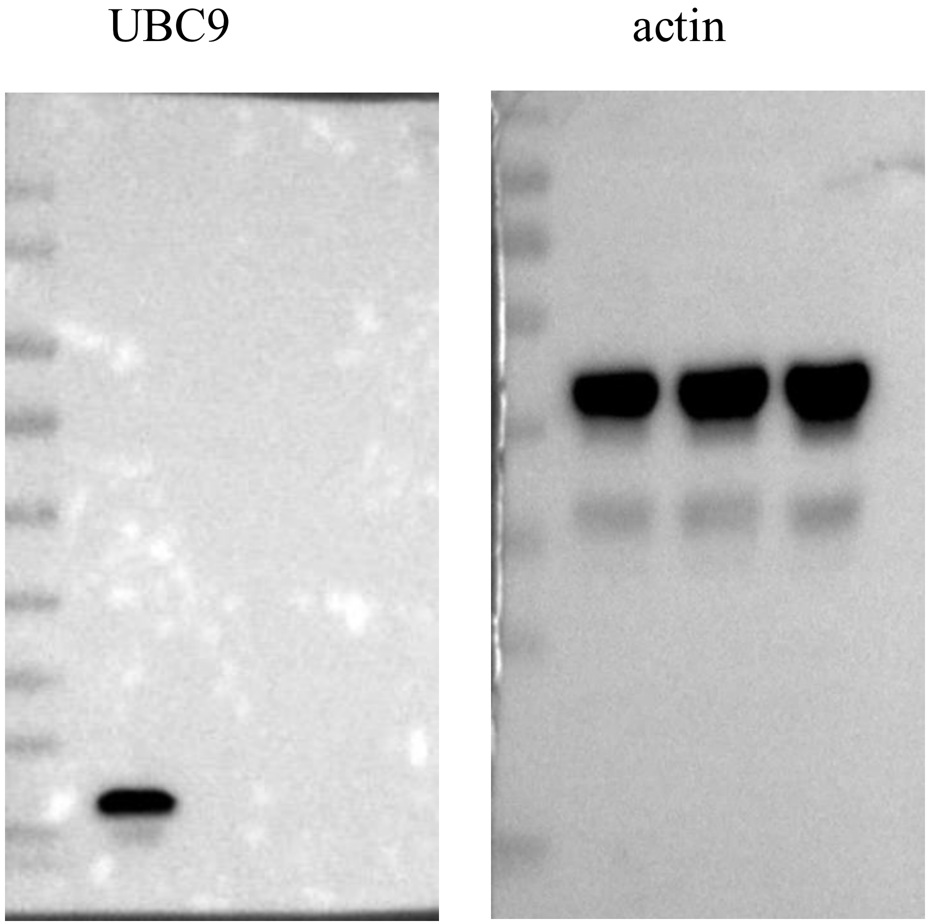

Figure3A

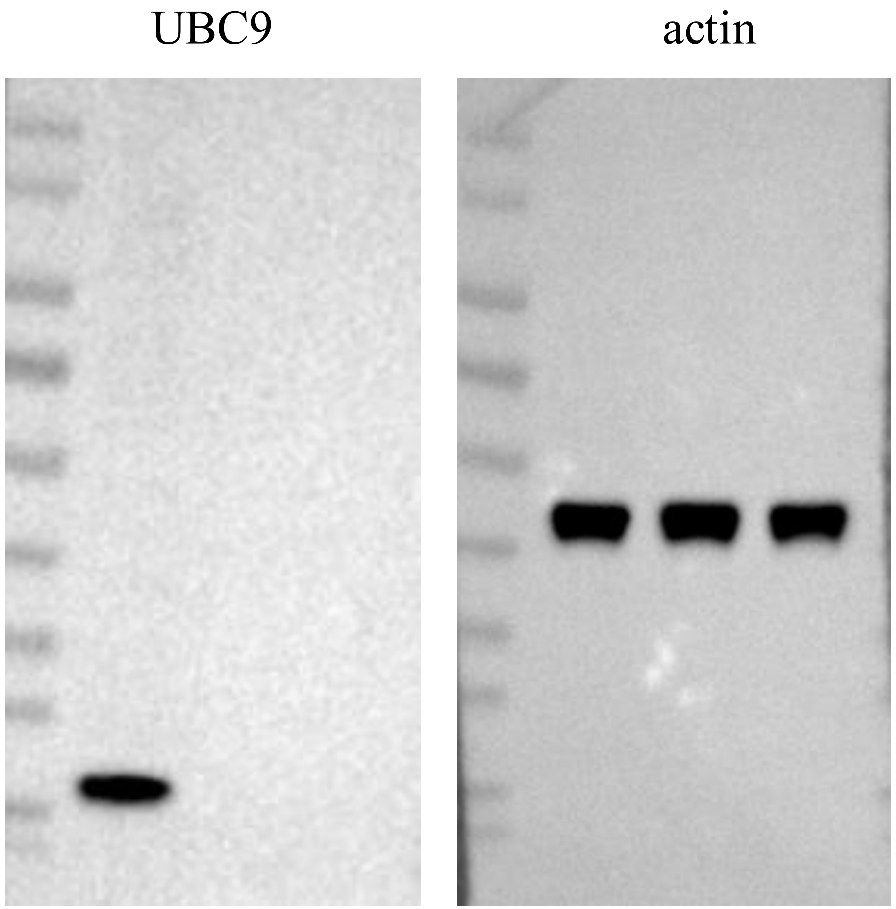

Figure4C-D

A549

PC9

UBC9 CORO1C UBC9 CORO1C

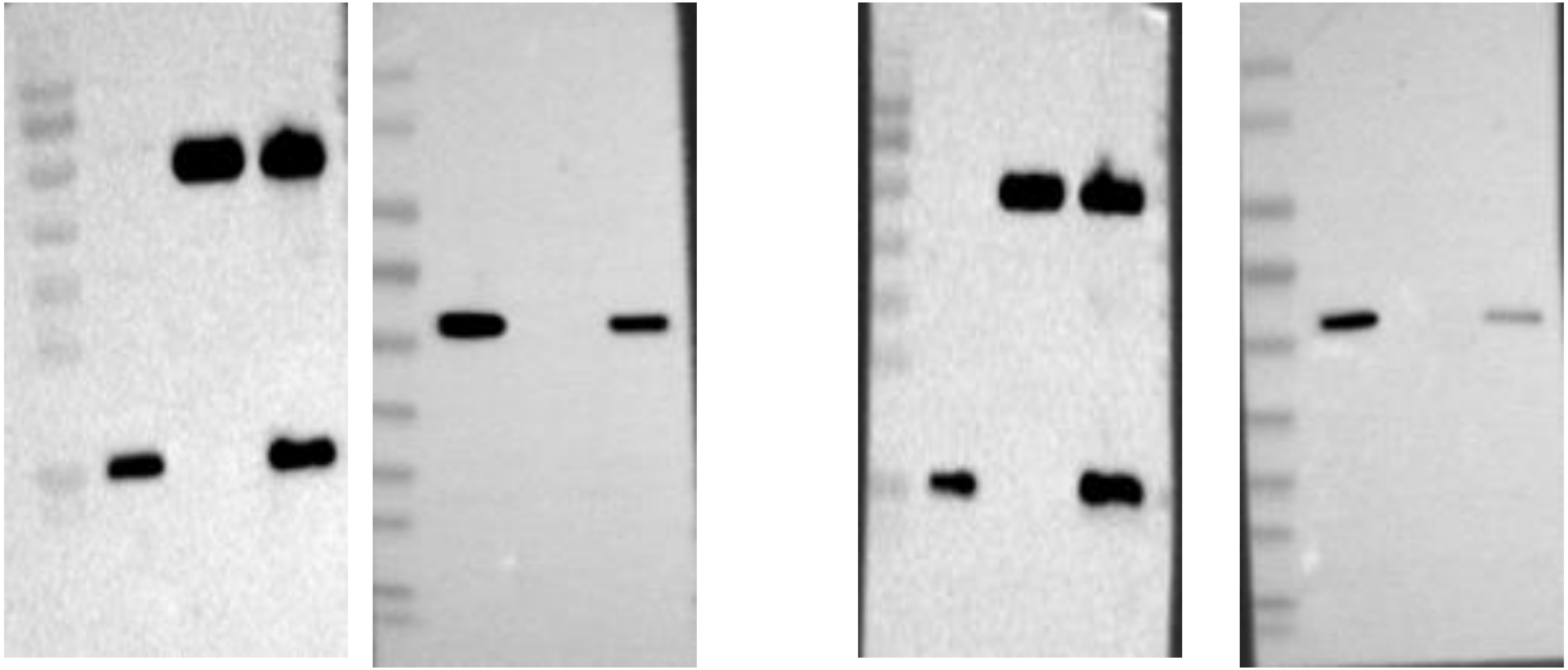

Figure4E

Figure4E IP

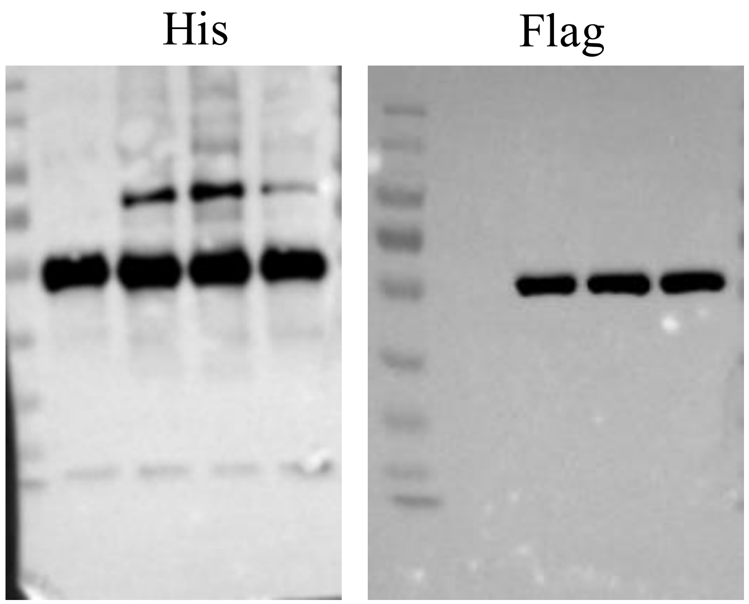

Figure4E

Figure4E Input

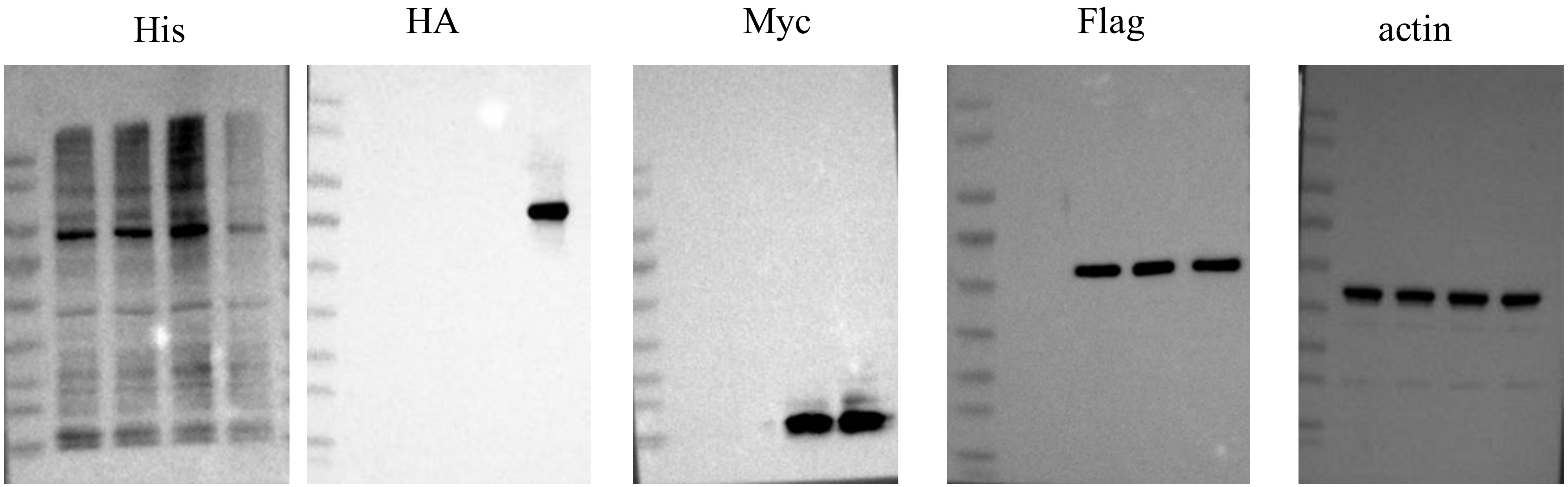

Figure4G

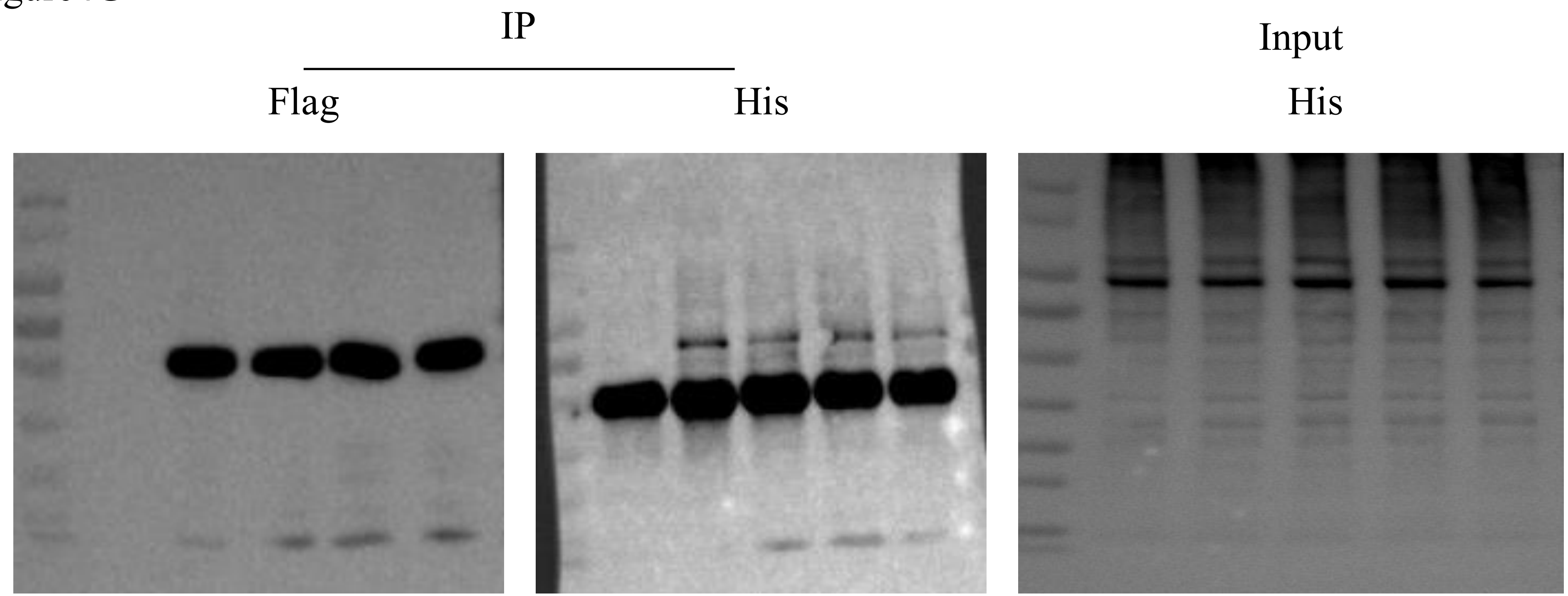

Figure4H

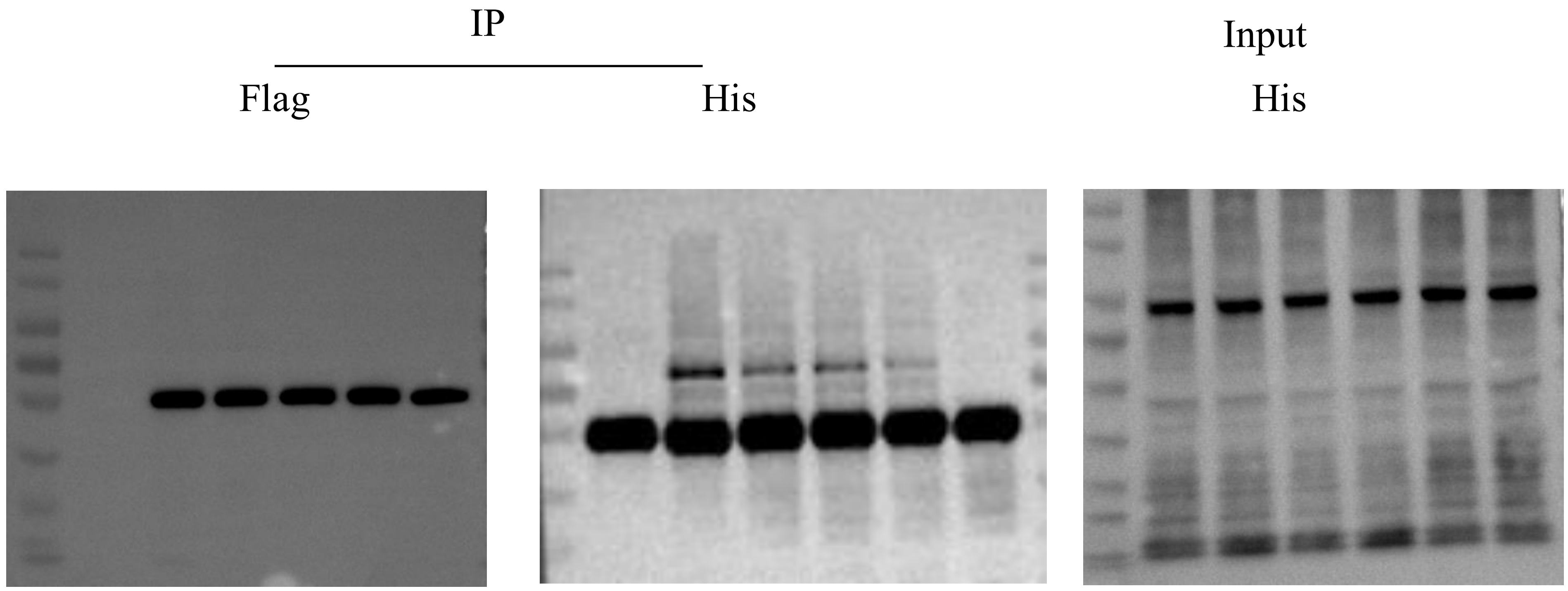

Figure6A

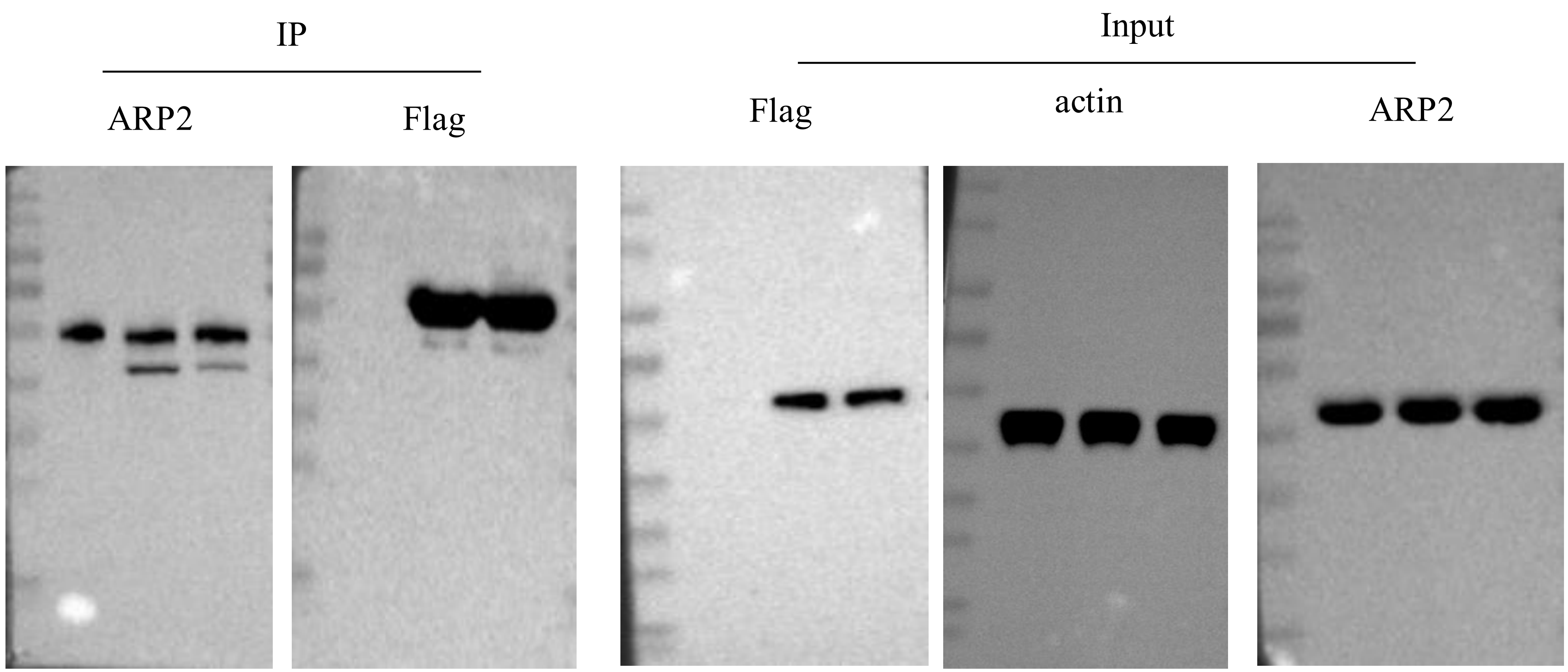

Supplement: Supplementary file 4 — Uncropped image [file 41419_2026_8653_MOESM4_ESM.pdf]
